# Combinations of motor measures more strongly predict adverse health outcomes in old age: the rush memory and aging project, a community-based cohort study

**DOI:** 10.1186/1741-7015-9-42

**Published:** 2011-04-20

**Authors:** Aron S Buchman, Sue E Leurgans,, Patricia A Boyle,, Julie A Schneider,, Steven E Arnold, David A Bennett

**Affiliations:** 1 Department of Neurological Sciences, Rush Alzheimer's Disease Center, Rush University Medical Center, 600 S. Paulina, Chicago, Illinois 60612, USA; 2 Department of Behavioral Science, Rush Alzheimer's Disease Center, Rush University Medical Center, 600 S. Paulina Street, Chicago, Illinois 60612, USA; 3 Department of Pathology, Rush Alzheimer's Disease Center, Rush University Medical Center, 600 S. Paulina Street, Chicago, Illinois 60612, USA; 4 Departments of Psychiatry and Neurology, Center for Neurobiology and Behavior, University of Pennsylvania, 125 South 31st Street, Philadelphia, Pennsylvania 19104, USA

## Abstract

**Objective:**

Motor impairment in old age is a growing public-health concern, and several different constructs have been used to identify motor impairments in older people. We tested the hypothesis that combinations of motor constructs more strongly predict adverse health outcomes in older people.

**Methods:**

In total, 949 people without dementia, history of stroke or Parkinson's disease, who were participating in the Rush Memory and Aging Project (a longitudinal community-based cohort study), underwent assessment at study entry. From this, three constructs were derived: 1) physical frailty based on grip strength, timed walk, body mass index and fatigue; 2) Parkinsonian Signs Score based on the modified motor section of the Unified Parkinson's Disease Rating Scale; and 3) a motor construct, based on nine strength measures and nine motor performances. Disability and cognitive status were assessed annually. A series of Cox proportional-hazards models, controlling for age, sex and education, were used to examine the association of each of these three constructs alone and in various combinations with death, disability and Alzheimer's disease (AD).

**Results:**

All three constructs were related (mean *r *= 0.50, all *P *< 0.001), and when considered individually in separate proportional-hazards models, were associated with risk of death, incident disability and AD. However, when considered together, combinations of these constructs more strongly predicted adverse health outcomes.

**Conclusions:**

Physical frailty, parkinsonian signs score and global motor score are related constructs that capture different aspects of motor function. Assessments using several motor constructs may more accurately identify people at the highest risk of adverse health consequences in old age.

## Introduction

Loss of motor function is a common consequence of old age, and is associated with adverse health consequences [1-5]. The specific motor abilities impaired in old age vary, and encompass a wide spectrum, including reduced gait speed and loss of muscle strength and bulk, balance, and dexterity [6-8]. Thus, the growing public-health challenge of identifying motor impairment in old age is complicated by the variability of its clinical expression.

Currently there is no single scale that can be used to assess motor impairments. Several constructs based on assessments of different motor abilities have been used to document motor impairments in old age, including presence of sarcopenia, based on muscle strength and bulk; [6] physical frailty, based on grip strength and gait speed; [7] the parkinsonian signs score, based on signs of bradykinesia, tremor, rigidity and parkinsonian gait [8]; and various summary motor measures based on testing a wider range of common motor performances [4,9]. Previous studies have linked these different measures with all-cause mortality, [1,10] incident disability [2-4] and dementia [5,11]. What remains unclear, however, is the extent to which these different motor constructs are overlapping or distinct, and whether combinations of these motor constructs might improve the prediction of adverse health outcomes. Thus, for example, in two participants with varying scores for parkinsonian signs, does considering their degree of physical frailty improve the prediction of adverse health outcomes? If true, this would suggest that these constructs have separate effects in predicting common adverse health outcomes. Further, such information would have important public-health consequences for identifying those older people at highest risk for adverse health consequences, and for delineating the full extent of the public-health challenge of motor impairment in old age.

We used data from 949 older people participating in the Rush Memory and Aging Project, a longitudinal study of common chronic conditions of aging, to examine the associations of three constructs that have been used in previous studies: physical frailty, parkinsonian signs score, and a composite motor score estimating the risk of death, incident disability and incident Alzheimer's disease (AD). We hypothesized that although all three constructs assess motor function, each construct captures aspects of motor abilities not appraised by the other constructs. Thus, we predicted that combinations of these three motor constructs would more strongly predict adverse health outcomes in older people compared with analyses using each motor construct alone.

## Methods

All participants were from the Rush Memory and Aging Project, which was conducted in accordance with the latest version of the Declaration of Helsinki, and approved by the institutional review board of Rush University Medical Center [12].

### Participants

Clinical evaluations for the study started in 1997, and at the time of these analyses, 1237 people had completed their baseline clinical evaluation. We excluded 95 people with dementia, 128 people with history of stroke, and 15 people with Parkinson's disease (PD) at baseline. Of the remaining 999 people, another 35 were excluded because follow-up was not possible (27 died before follow-up, and eight had not been in the study long enough for follow-up). This left 964 participants at baseline who were eligible for follow-up, and 949 (98.4%) of these had completed one or more follow-up evaluations. These participants had an average of nearly five follow-up examinations (mean ± SD 4.7 ± 2.4). The participants' baseline characteristics are shown in Table 1.

**Table 1:** Characteristics of the cohort at baseline

Variable	**Results**^**a**^
Age, years	79.7 ± 7.3
Women, *n *(%)	704 (74.2)
White non-Hispanic, *n *(%)	837 (88.2)
Education, years	14.5 ± 3.2
MMSE^b^, marks out of 30	27.9 ± 2.0
Global Cognition, composite	0.11 ± 0.53
Body mass index	27.7 ± 5.31
Physical activity, hours/week	3.2 ± 3.53
Existing illnesses, %	
Myocardial infarction	11.3
Congestive heart failure	5.2
Claudication	6.7
Diabetes	13.7
Smoking	40.3
Hypertension	58.5

^a^Data are mean ± SD unless otherwise indicated.

^b^Mini Mental Status Examination.

### Assessment of clinical diagnoses

Subjects underwent a uniform structured clinical evaluation including medical history, clinical examination, and cognitive performance testing as previously described [12]. Clinical diagnoses were made using a multistep process [12]. Participants were evaluated in person by an experienced clinician, who diagnosed dementia, stroke and PD using commonly accepted procedures [13-15].

### Assessment of physical frailty

Physical frailty is an evolving concept, with numerous definitions proposed. The categorical measure proposed by Fried *et al*. is a widely used construct [7]. We modified this measure in two ways. First, physical activity may be an important cause (or consequence) of impaired motor function, therefore we modified the physical frailty construct as proposed by Fried *et al*. to remove physical activity, in order to examine its association with physical frailty and other constructs related to motor abilities [16-18]. Second, rather than using a categorical measure, we constructed a continuous composite measure of physical frailty. Composite measures have been used effectively in other areas of aging research [19], and offer considerably more power to detect associations and to document change over time [1,20]. As described previously, composite physical frailty correlates strongly with the frailty measure used by other investigators. Furthermore, in previous publications, we have demonstrated the predictive validity of composite physical frailty, illustrating its association with a wide range of adverse health outcomes including mortality, incident disability, incident myocardial infarction, incident AD and cognitive decline, and it has been used to document the rate of change in physical frailty [1,11,21]. Physical frailty in this study was based on grip strength and timed walk for 8 feet, while body composition was based on body mass index and fatigue [22]. Fatigue was assessed with two questions from a modified version of the Center for Epidemiologic Studies-Depression (CES-D) Scale. Composite physical frailty was constructed by converting the raw score from each of the four component measures to z scores using the mean and standard deviation from all participants at baseline, as described previously [1,7,11,20].

### Assessment of parkinsonian signs

A modified form of the motor section of the Unified Parkinson's Disease Rating Scale (mUPDRS) was used for each patient. This assesses four parkinsonian domains (bradykinesia, rigidity, parkinsonian gait and tremor) as previously described [12]. Each sign was scored from 0 to 100, and a global parkinsonian signs score was obtained by averaging the scores of the four individual parkinsonian domains [23].

### Assessment of Global Motor Score

Portable hand-held dynamometers (Manual Muscle Test System, Model 01163; Lafayette Instruments, Lafayette, IN, USA) were used to assess arm abduction, flexion and extension, hip flexion, knee extension, plantar flexion, and ankle dorsiflexion. Grip and pinch strength were measured bilaterally using a hydraulic dynamometer (Jamar^®^; Lafayette Instruments). Motor performances were tested in both arms and legs, using seven performance-based tests of leg function and two tests of motor performance of the arms, as previously described [9].

We scored each of the performance measures so that higher scores were associated with better performance. To ensure the same directionality in all the performance measures, we first reciprocated the recorded values for five variables (the time and number of steps for the walking and turning tasks, and the number of steps off the line for tandem walking). A score of 0 was recorded if a participant was unable to perform a particular task. Each score was then scaled by dividing by the sex-specific median value of the non-zero values at baseline [24-26]. The scaled scores for each measure were then averaged to obtain a composite global motor score for each subject. In previous publications from this cohort, measures were centered before scaling when calculating an alternative global motor score using *z *scores [9]. The correlation of our global motor score with the previously published score was high (*r *= 0.89, *P *< 0.001).

### Assessment of disability

Basic activities of daily living were assessed using a modified version of the Katz Index, a self-report measure that assesses six activities: feeding, bathing, dressing, toileting, transferring, and walking across a small room [27]. Participants who reported a requirement for help or an inability to perform one or more tasks were classified as disabled.

### Comorbidities and other covariates

Demographic information including date of birth, gender and years of education were collected via participant interview. The number of hours of physical activity per week was assessed using questions adapted from the 1985 National Health Interview Survey [16]. We summarized vascular risk factors (hypertension, diabetes mellitus and smoking) and vascular disease burden (myocardial infarction, congestive heart failure and claudication), as previously described [28].

### Mortality

Participation in the Memory and Aging Project includes the patient agreeing to donation of brain, spinal cord, and selected muscles and nerves at the time of death, and the study has an autopsy rate of >80%. At the time of these analyses, vital status was available for all participants.

### Statistical analyses

Pearson correlations were used to examine the bivariate associations between motor constructs and demographic variables, and *t*-tests were used to compare baseline characteristics. Statistical significance was considered to be *P *< 0.05.

The goal of these analyses was to test the hypothesis that models using combinations of the three motor constructs would provide additional explanatory power for the health outcome of interest compared with models using the individual motor constructs alone. We used a set of eight proportional-hazards models to estimate risk of death associated with demographic variables (age, sex and education), and with the three motor constructs alone and in combination.

**Figure 1:**
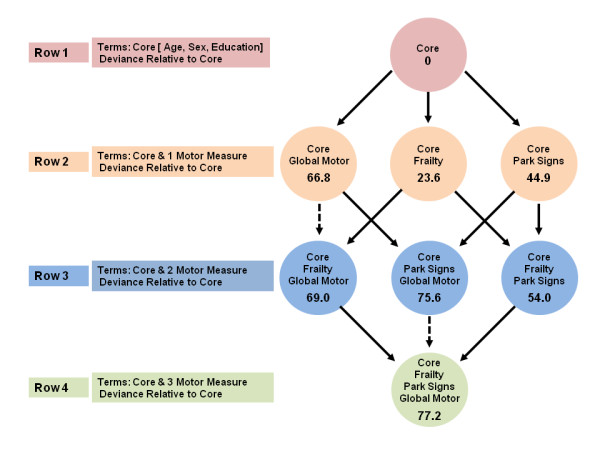
**Motor constructs and risk of death**. We examined the associations of three motor constructs with the risk of death in a set of seven proportional-hazards models that all controlled for age, sex and education. This set comprised three models that included terms for each of the three motor constructs alone, and four models that included combinations of two or more motor constructs. Each model is represented by a circle that shows the terms included in the model, and the model deviance relative to the core model, which included only terms for age, sex and education. A deviance test comparing two models that differ by one variable was significant (α = 0.05) if the deviances from the core model differed by >+3.84. In this figure, a solid line denotes a significant reduction (that is, a difference of > + 3.84), and a dotted line denotes a non-significant reduction (that is, a difference < + 3.84).

First, we determined the contribution of the demographic variables age, gender and education to the risk of death (core model) (Figure 1, row 1). Then in three separate models, each of which controlled for these demographic variables, we included each of the motor constructs alone to estimate its independent association with risk of death (Figure 1, row 2). In further analyses, we examined three models that were different combinations of two of the three motor constructs (Figure 1, row 3), and in a final model we examined all three motor constructs together in a single model (Figure 1, row 4)

Using the Wilks or deviance test, we compared the three models of each of the motor constructs alone with the four models consisting of combinations of these motor constructs. We chose this approach because Wald's method for obtaining observed significance levels for terms in a proportional-hazard model can result in *P*-values that are spuriously large. The deviance tests are extensions of partial *F *tests to generalized linear models [29]. This approach allowed us to compare two models directly, with model (M)1 being nested in M2. That is, M2 consists of the variables in M1 plus *k *additional variables. The coefficients are estimated by maximizing a partial likelihood function, and the maximum of the likelihood function for M2 will always be larger than that for M1. Then, under the null hypothesis that M1 is the true model, twice the difference of the logarithm of the likelihood for the two models (a statistic known as the deviance between the two models), follows an approximate χ^2 ^distribution with *k *degrees of freedom. If M1 is not the true model, the deviance test will tend to be larger.

These different comparisons are summarized in Figure 1. We compared three pairs of models in which M1 was the core model (Figure 1, row 1) with only age, sex and education included, and M2 added exactly one motor construct (Figure 1, row 2). We then compared three pairs of models in which M1 was one of the models with a single motor construct from the previous set and M2 added asecond motor construct (Figure 1, row 3). For these three deviance tests, the deviance between a model in row 2 and one in row 3 is the difference between the corresponding deviances from the core. Finally, we compared three more models in which M2 contained all three motor constructs (Figure 1, row 4) and M1 omitted one of these three constructs (Figure 1, row 3). The deviance tests of these comparisons are the differences of the deviance from the core to the row 4 model and the deviances from the core to the row 3 models. In these analyses, when comparing two models, a model deviance (that is, numbers shown in circles in Figure 1) of >3.84 was significant. Thus, models associated with a larger model deviance supported the hypothesis that combinations of motor constructs provide additional explanatory power for the health outcome of interest. An additional advantage of this approach is that examining these combinations together permits determination of how the order of introduction of terms for motor constructs can affect the strength of the associations with the respective adverse health outcomes.

**Figure 2:**
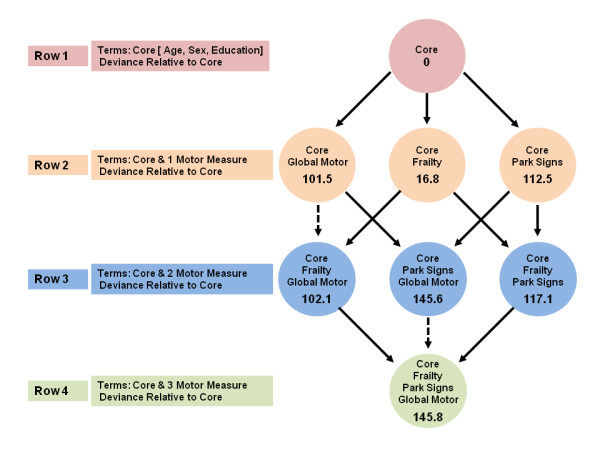
**Motor constructs and incident disability**. We examined the associations of three motor constructs with incident disability in a set of seven proportional-hazards models that all controlled for age, gender and education. This set comprised three models that included terms for each of the three motor constructs alone, and four models that included combinations of two or more motor constructs. Each model is represented by a circle that shows the terms included in the model, and the model deviance relative to the core model, which included only terms for age, sex and education. A deviance test comparing two models that differ by one variable was significant (α = 0.05) if the deviances from the core model differed by > + 3.84. In this figure, a solid line denotes a significant reduction (that is, a difference of > + 3.84), and a dotted line denotes a non-significant reduction (that is, a difference < + 3.84).

**Figure 3:**
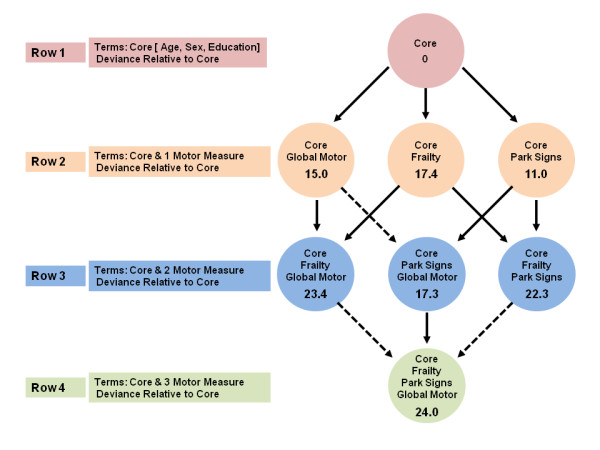
**Motor constructs and incident Alzheimer's disease (AD)**. We examined the associations of three motor constructs with incident AD in a set of seven proportional-hazards models that all controlled for age, sex and education. This set comprised three models that included terms for each of the three motor constructs alone, and four models that included combinations of two or more motor constructs. Each model is represented by a circle that shows the terms included in the model, and the model deviance relative to the core model, which included only terms for age, sex and education. A deviance test comparing two models that differ by one variable was significant (α = 0.05) if the deviances from the core model differed by >+3.84. In this figure, a solid line denotes a significant reduction (that is, a difference of >+3.84), and a dotted line denotes a non-significant reduction (that is, a difference < + 3.84).

We used a similar series of models for discrete (tied) data to examine the relationships of the three motor constructs with the risk of developing incident disability (Figure 2) and then a third series for the risk of developing incident AD (Figure 3).

Grip strength and timed walk were components common to both global motor score and physical frailty, thus to avoid duplication, we removed these two components from the global motor score before analyzing combinations of these constructs. In a final analysis, we added back the two components of grip strength and timed walk that had been removed from the global motor score measure (see above), and reran the set of models above.

Models were examined graphically and analytically, and assumptions were judged to have been met adequately. The models for this paper were programmed using SAS/STAT software (version 9.1.3 of the SAS^® ^System for Linux; SAS Institute Inc., Cary, NC, USA) [29].

## Results

### Descriptive properties of physical frailty, parkinsonian signs score parkinsonian signs score, global motor score and their inter-relationships

Physical frailty ranged from -1.71 to 1.90 (mean ± SD -0.054 ± 0.55), with higher scores indicating a greater degree of physical frailty or impairment. Physical frailty was related to age (*r *= 0.33, *P *< 0.001) and education (*r *= -0.21, *P *< 0.001), and men were more frail than women (*t *= 11.80 [945], *P *< 0.001).

Parkinsonian signs score ranged from 0 to 49 (mean ± SD 8.46 ± 6.88), with higher scores indicating more parkinsonian signs. The score was related to age (*r *= 0.33, *P *< 0.001) and education (*r *= -0.10, *P *= 0.002) and did not differ by sex (*t *= 0.23 [947], *P *= 0.82).

Global motor score ranged from 0.16 to 2.03 (mean ± SD 1.01 ± 0.27), with higher scores indicating better motor function. Global motor score was related to age (*r *= -0.50, *P *< 0.001) and education (*r *= -0.11, *P *< 0.001), and did not differ by gender (*t *= -0.91 [947], *P *= 0.36).

As expected, the three constructs were all related to one another. Physical frailty was related to both parkinsonian signs score (*r *= 0.37, *P *< 0.001) and global motor score (*r *= -0.51, *P *< 0.001). Parkinsonian signs score was also related to global motor score (*r *= -0.63, *P *< 0.001).

### Physical frailty, parkinsonian signs score parkinsonian signs score, global motor score and risk of death

Over an average follow-up of >5 years (mean ± SD 5.51 ± 2.37 years; range 0.8 to 11.6), 253 participants (26.7%) died. In separate proportional-hazard models controlling for age, sex and education, we found that physical frailty, parkinsonian signs score parkinsonian signs score and global motor score were all individually associated with risk of death (Table 2). For example, a 0.6-unit (about 1 SD) increase in the level of physical frailty at baseline was associated with a risk of death approximately 1.5-fold higher (Table 2); a 7-unit (1 SD) increase in the level of parkinsonian signs score at baseline was also associated with a risk of death approximately 1.5-fold higher, and a 0.27-unit (1 SD) decrease in the level of global motor score at baseline was associated with a twofold higher risk of death.

**Table 2:** Motor constructs and risk of adverse health outcomes^a^

Predictor	Death, HR (95% CI)	AD^b^, HR (95% CI)	Disability, HR (95% CI)
Frailty	1.48 (1.27-1.73)	1.56 (1.27-1.92)	1.44 (1.21-1.71)
PSS^c^	1.53 (1.36-1.72)	1.35 (1.14-1.59)	2.20 (1.90-2.53)
GMS^d^	0.51 (0.42-0.60)	0.62 (0.49-0.78)	0.39 (0.32-0.47)

^a^Based on proportional-hazard models that controlled for age, sex and education for each of the three outcomes. Results show the effect for 1 SD unit higher level of physical frailty (1 SD = 0.6 units), parkinsonian sign score (1 SD = 7 points) and global motor score (1 SD = 0.27 units). Higher baseline values for physical frailty and parkinsonian signs score (that is, poorer performance) were associated with an increased risk of adverse health outcomes (HR >1.00) whereas a higher baseline value for global motor score (that is, better performance) was associated with a decreased risk of adverse health outcomes (HR < 1.0).

^b^Alzheimer's disease.

^c^Global Motor Score.

^d^Parkinsonian Signs Score.

Another way of expressing the magnitude of the risk of death associated with a higher level of baseline physical frailty is to compare the estimate for physical frailty with that of age. As noted above, age at baseline was also associated with an increased risk of death (hazard ratio (HR) for age = 1.10; 95% CI 1.07-1.12), resulting in about a 10% increase in the risk of death for each year of age at baseline. Comparison of the estimates for physical frailty and age showed that the risk of death associated with a 0.6-unit (1 SD) increase in the level of physical frailty at baseline was comparable with a participant being >4 years older at baseline (physical frailty estimate of 0.392 versus age estimate of 0.092). A similar comparison would show that a seven-point (1 SD) increase in parkinsonian signs score at baseline was comparable with the risk associated with being about 5 years older at baseline, and a 0.27-unit (1 SD) decrease in global motor score was comparable with being 9 years older at baseline.

We examined a set of proportional-hazards models that included all possible combinations of these three constructs to determine if the combinations with two or more constructs showed a significant reduction in model deviance (that is, a stronger association with risk of death) compared with the individual constructs alone. In these analyses (Figure 1), a significant model deviance when one variable is added to a model corresponded to a deviance test statistic of >3.84. In Figure 1, a solid line denotes a deviance >3.84 and a dotted line denotes a deviance <3.84, and the addition of a term for any one of the three constructs to the core model (having the terms for age, sex and education) produced a significant deviance statistic (Figure 1, rows 1 to 2). Addition of a term for physical frailty did not improve fit if global motor score was already included in the model (Figure 1, rows 2 to 3, dotted arrows). By contrast, other pairs of motor measures (global motor score plus parkinsonian signs score, and physical frailty plus parkinsonian signs score), produced significant improvements in model fit (Figure 1, rows 2 to 3, solid arrows). Finally, when all three terms were considered together (Figure 1, row 4), the combination of parkinsonian signs score and global motor score without physical frailty (Figure 1, row 3) was the 'best' model for predicting the risk of death.

In further analyses, we added back grip strength and timed walk, which had been removed from the global motor score measure, and the results were unchanged (results not shown).

### Physical frailty, parkinsonian signs score parkinsonian signs score, global motor score and incident disability

We restricted the next analysis to the 841 people (88.6% of 949 people) without Katz disability at baseline. Over an average follow-up of almost 5 years (mean ± SD 4.9 ± 2.39 years), 295 of 841 participants (35.1%) developed Katz disability. We examined the associations of each of these three constructs with incident disability by fitting a set of proportional-hazard models that controlled for age, sex and education. Physical frailty, parkinsonian signs score and global motor score were all associated with incident disability (Table 2). For example, a 0.6-unit (about 1 SD) higher level of physical frailty at baseline was associated with about a 1.4-fold higher risk of disability (Table 2). A 7-point (1 SD) higher level of parkinsonian signs score at baseline was associated with an approximately twofold higher risk of disability, and a 0.27-unit (1 SD) lower level of global motor score at baseline was associated with a >2.3-fold higher risk of disability (Table 2).

In subsequent analyses, the combinations of global motor score plus parkinsonian signs score or physical frailty plus parkinsonian signs score gave an improved fit when included together in the same models (Figure 2, rows 2 to 3, solid arrows) compared with models of the individual constructs alone (Figure 2, row 2). As with risk of death, the addition of a term for physical frailty was not significant if global motor score was already in the model (Figure 2, dotted arrows). Thus, both forward and backward selection suggested that the combination of parkinsonian signs score and global motor score (Figure 2, row 3) together are the 'best' model for predicting risk of incident disability.

In further analyses, we added back grip strength and timed walk, which had been removed from the global motor score measure, and the results were unchanged (results not shown).

### Physical frailty, parkinsonian signs score parkinsonian signs score, global motor score and risk of AD

During an average follow-up of >4 years (mean ± SD 4.2 ± 2.35), 155 of the 919 participants (16.9%) developed AD. We examined the associations of each of the three motor constructs with the risk of AD by fitting a set of proportional-hazard models that controlled for age, sex and education. Physical frailty, parkinsonian signs score and global motor score were each associated with risk of AD (Table 2). For example, a 0.6-unit (1 SD) higher level of physical frailty at baseline was associated with a 1.5-fold higher risk of AD; a 7-point (1 SD) higher level of parkinsonian signs score at baseline was associated with about a 1.3-fold higher risk of AD (Table 2), and a 0.27-unit (1 SD) lower level of global motor score at baseline was associated with a 1.6-fold higher risk of AD (Table 2).

In subsequent analyses, the combinations of global motor score plus physical frailty or physical frailty plus parkinsonian signs score gave a significantly improved fit when included together in the same model (Figure 3, rows 2 to 3, solid arrows). Parkinsonian signs score was not significant if global motor score was already in the model (Figure 3, rows 2 to 3, dotted arrow). In a final model, we considered all three constructs together. In contrast to the results for risk of death and incident disability, global motor score was not significant if physical frailty and parkinsonian signs score were already in the model (Figure 3, rows 3 to 4). Thus, although the combinations of these constructs predicted incident AD more strongly, the combination of physical frailty with either parkinsonian signs or global motor score did not produce a better fit.

In further analyses, we added back grip strength and timed walk, which had been removed from the global motor score measure, and the results were unchanged (results not shown).

## Discussion

In a cohort of 949 older people without dementia, history of stroke or PD, we found that three well-studied motor constructs - physical frailty, parkinsonian signs score and global motor score - were all associated with adverse health consequences including mortality, incident disability and incident AD. Furthermore, when considered together in the same models, combinations of these three constructs substantially improved the prediction of adverse health outcomes relative to the constructs considered alone. Thus, these three related motor constructs capture, in part, different aspects of motor function, therefore assessments using more than one motor measure might more accurately identify older people at risk for adverse health consequences.

Loss of motor function in old age is common, and is associated with adverse health consequences. There are currently about 40 million people over the age of 65 years in the USA, and by 2030, this will have increased to more than 70 million [30]. It is estimated that 40% or more of these people may have some elements of motor impairment by the age of 80 years [31]. The growing public-health concern with motor impairment in old age is even more challenging because of the wide variation in the specific motor abilities that may be impaired. For example, one or more of the following are commonly seen in older people: loss of muscle strength and bulk, loss of balance, loss of dexterity and reduction in gait speed [6-8]. Currently, there is no single, universally accepted scale for documenting motor impairment in old age. Thus, efforts to document motor impairment in older people have been subsumed under several different constructs, often developed by different disciplines within the field of aging, which are each based on the assessments of different motor abilities [6-9]. Previous studies have examined each of these constructs alone, and reported that these various motor constructs are associated with adverse health outcomes including mortality, disability and AD [6-9].

The current study extends on previous findings in several important ways. First, in contrast to previous studies, which usually focused on a single construct, the current study examined three well-studied constructs in the same population, and confirmed that when each of these three constructs is considered alone in separate models, they each predict the likelihood of death, disability and AD. Second, direct comparison showed that these three constructs are related to one another. Thus, reports about use of these three constructs in old age should be considered related literature that inform on one another. Finally, the current study showed that combinations of two or more of these constructs had separate effects on adverse health outcomes, and that the associations varied by outcome. For example, parkinsonian signs score and global motor score were most strongly related to risk of death and incident disability, whereas physical frailty and either of the other two constructs most strongly predicted incident AD. Together, these findings have important translational consequences for the assessment of motor impairments in old age, because they suggest that a battery of motor measures may be necessary to understand age-related motor decline more fully and to identify more accurately the people at risk for negative health outcomes in our rapidly aging population.

Localized brain lesions (for example, stroke), specific diseases (for example, PD, radiculopathy or myositis) and localized musculoskeletal disease (for example, osteoarthritis) may selectively impair distinct motor abilities while sparing others [32-34]. These dissociations suggest that motor function is not a unitary process, and that the clinical presentations of motor impairment vary with the localization of central nervous system and musculoskeletal system dysfunction [35-38]. Because the three different constructs examined in this study assess different aspects of motor function, it is not surprising that they showed different associations with health outcomes. Further work is needed to determine if comorbidities such as vascular risk factors or diseases may have differential effects on these constructs.

The findings in the current study are supported by recent advances in neuroscience, which have begun to characterize the complex brain processes necessary to ensure accurate movements. Integration of a wide range of sensory and visuospatial information (for example, postural control, spatial navigation and joint position) is essential for accurate movements [39], and different regions within motor-related brain regions may control distinct aspects of movement (for example, speed versus balance) [40]. Finally, brain structures outside traditional motor regions are also crucial for movements [41,42]. Translating these basic scientific observations into the clinical domain will require the development of a battery of clinical motor measures that reflect the various neural and non-neural mechanisms underlying different motor abilities. Future research should determine if our findings are due to differences in the underlying biologic substrate of the different constructs, as this would suggest that different approaches are needed to treat and prevent different motor impairments.

Several factors increase confidence in the findings from this study. Cognitive and motor functions were evaluated as part of a uniform clinical evaluation, and incorporated three widely accepted motor measures which are reliable and available. Strength testing was performed for all four limbs, and motor performances were tested in both the arms and the legs. The aggregation of multiple measures of motor function into composite motor measures is likely to yield a more stable measure of motor function, and may increase the power to identify adverse health consequences of motor decline in aging. In addition, a relatively large number of older people were studied, with high rates of follow-up participation, so that there was adequate statistical power to identify the associations of interest while controlling for potentially confounding demographic variables and reducing bias.

The limitations of this study include the selected nature of the cohort, thus replication of these results in a population-based study is important. Further, although the findings from this study suggest that assessment of motor function may have clinical implication, additional work is needed to determine which measures are most clinically relevant. Finally, because people with stroke or PD at baseline were excluded, the results almost certainly underestimate the magnitude of the association between motor function with adverse health outcomes.

## Conclusions

In a cohort of nearly 1000 community-dwelling older people free of dementia, PD or history of stroke, who were followed up for up to 12 years, we found that three different motor constructs (physical frailty, parkinsonism and a composite measure of motor performance measures) were all related, and were all associated with death and the subsequent development of disability and AD. Most importantly, these results extend those of previous studies by showing that combinations of these three constructs in the same models more strongly predict adverse health outcomes than the individual constructs alone. Thus, a battery of motor measures may be necessary to understand age-related motor impairments more fully, and thereby identify at-risk people more accurately in order to design interventions that decrease the growing burden of motor impairment in our rapidly aging population.

## Competing interests

The authors declare that they have no competing interests.

## Authors' contributions

ASB had full access to all the data in the study, and takes responsibility for the integrity of the data and the accuracy of the data analysis, and affirms that everyone who contributed significantly to the work has been listed. ASB was involved with study concept and design, analysis and interpretation of data and preparation of the manuscript. SEL, PAB, JAS, SEA and DAB were involved in study concept and acquisition of data, assisted with the analysis and interpretation of the data, and critically revised the manuscript for important intellectual content. All authors have seen and approved the final version.

## Pre-publication history

The pre-publication history for this paper can be accessed here:

http://www.biomedcentral.com/1741-7015/9/42/prepub
